# The Relationship Between How Participants Articulate Their Goals and Accomplishments and Weight Loss Outcomes: Secondary Analysis of a Pilot of a Web-Based Weight Loss Intervention

**DOI:** 10.2196/41275

**Published:** 2023-03-16

**Authors:** Danielle E Jake-Schoffman, Molly E Waring, Joseph DiVito, Jared M Goetz, Cindy Pan, Sherry L Pagoto

**Affiliations:** 1 Department of Health Education and Behavior College of Health and Human Performance University of Florida Gainesville, FL United States; 2 Center for Integrative Cardiovascular and Metabolic Disease University of Florida Gainesville, FL United States; 3 Department of Allied Health Sciences University of Connecticut Storrs, CT United States; 4 UConn Center for mHealth & Social Media University of Connecticut Storrs, CT United States

**Keywords:** weight loss, social media, goal setting, web-based program, behavior change, habit formation, diabetes, Facebook, lifestyle

## Abstract

**Background:**

In behavioral weight loss interventions, participants are asked to set weekly goals to support long-term habits that lead to weight loss. Although participants are asked to set and accomplish weekly goals, we do not know how often they do this and whether doing so is associated with weight loss. Web-based weight loss interventions allow for the analysis of participant engagement data, including how participants articulate their goals and accomplishments.

**Objective:**

Using engagement data from a web-based weight loss intervention, we examined whether participants articulating their goals and accomplishments in measurable and repeating terms were associated with greater weight loss.

**Methods:**

Adults with overweight or obesity received a 12-week Facebook-delivered weight loss intervention based on the Diabetes Prevention Program Lifestyle Intervention. Participants replied to conversation threads that queried about their goals and accomplishments. Two independent coders classified participants’ posts that articulated goals or accomplishments as measurable or repeating. Crude and age-adjusted linear regression models were used to examine the relationship between the frequency of post type and percent weight loss.

**Results:**

Participants (N=53; n=48, 91% female; n=48, 91% non-Hispanic White) were on average 46.2 (SD 10.5) years old with a mean BMI of 32.4 (SD 4.8) kg/m^2^. Over 12 weeks, participants shared a median of 4 (IQR 1-8) posts that reported goals and 10 (IQR 4-24) posts that reported accomplishments. Most participants shared ≥1 post with a goal (n=43, 81%) and ≥1 post with an accomplishment (n=47, 89%). Each post reporting a goal was associated with 0.2% greater weight loss (95% CI −0.3% to 0.0%). Sharing ≥1 post with a repeating goal was associated with an average of 2.2% greater weight loss (95% CI −3.9% to −0.4%). Each post with a repeating goal was associated with an average of 0.5% greater weight loss (95% CI −1.0% to 0.0%). Sharing ≥1 post with measurable and repeating goals was associated with an average of 1.9% greater weight loss (95% CI −3.7% to −0.2%). Sharing each post with an accomplishment was associated with an average of 0.1% greater weight loss (95% CI −0.1% to 0.0%). Every post with an accomplishment that was repeating was associated with an average of 0.2% greater weight loss (95% CI −0.3% to 0.0%). Sharing other types of goals and accomplishments was not associated with weight loss.

**Conclusions:**

In a web-based weight loss intervention, stating goals in repeating or both measurable and repeating terms was associated with greater weight loss, but simply stating them in measurable terms was not. For accomplishments, only those articulated in repeating terms were associated with greater weight loss. Posts about one-time goals and accomplishments represent an opportunity to encourage planning for future behaviors. Future research should examine if stating goals and accomplishments in repeating terms signals habit formation.

## Introduction

Nearly 40% of US adults have obesity [1], putting them at risk for chronic diseases including diabetes [2], heart disease [3], and some cancers [4]. Behavioral interventions are effective at reducing these risks. For example, the Diabetes Prevention Program (DPP) Lifestyle Intervention [5] has been shown to produce significant weight loss and reduce the risk of diabetes [6]. However, behavioral interventions have not been widely disseminated in part due to the high cost [7] and barriers such as scheduling and transportation issues [8,9]. Technology tools have the potential to expand the reach of behavioral interventions through increased accessibility and access.

Behavioral weight loss programs that have traditionally been delivered in-person have now been adapted for web-based delivery [10] through novel platforms, including commercial social media platforms [11]. A recent systematic review of 21 studies of technology-delivered interventions based on the DPP Lifestyle Intervention found promising weight loss efficacy [10]. Engagement in web-based interventions entails participant actions like viewing content, commenting on posts, posting, and reacting to posts (eg, hitting a like button). In general, engagement in web-based weight loss interventions is variable, ranging from <1 to >100 mean posts made per participant [12]. However, greater participant engagement is generally associated with more weight loss [12-15]. Less is known about the type and quality of engagement that has the most impact on weight loss. As a first step in this line of inquiry, the relationship between overall participant post counts and broad categories of posts with weight loss was examined [16] in a Facebook-delivered weight loss intervention. We found that while the overall volume of participant posts was associated with weight loss, not all types of participant posts were predictive of weight loss [16]. Specifically, participant posts that mentioned a goal or an accomplishment were associated with weight loss, but posts reporting problems losing weight or that simply served to support other group members were not [16].

Posting more about goals and accomplishments, which are predictive of weight loss, is not surprising because goal setting is an effective behavioral strategy. Participants in the DPP are coached on setting weekly “SMART” (Specific, Measurable, Action-oriented, Relevant, and Time-bound) goals, which are goals that are specific, measurable, attainable, relevant, and time-bound [17] and following up on them effectively [5,18]. SMART goals may more effectively lead to habit formation than goals without these parameters. A goal is considered measurable if it is articulated in a way that can be quantified. For example, an exercise goal that mentions frequency, duration, or intensity would be considered measurable. A goal is considered time-bound if it specifies when the action is going to occur. Ideally, time-bound goals specify a repeating habit as opposed to a one-time action. For example, an exercise goal that mentions a frequency greater than 1 episode is more likely to develop into a habit than an exercise goal that mentions a frequency of 1 episode (eg, “I am going to work out 3 days this week” vs “I am going to work out today”) [19]. Habit formation theory suggests that bridging the intention-translation gap, or the disconnect between the desire to routinize new healthy behaviors and actually doing so, might be overcome in part with goal setting that involves careful planning and tracking progress [18]. Thus, the articulation of goals in measurable and repeating ways is likely to be associated with better outcomes. Similarly, the articulation of accomplishments in measurable and repeating ways may also be associated with better outcomes because this would also seem to be evidence of habit formation. Articulation of accomplishments in specific terms may also be related to successful feedback loops in the goal-setting process, whereby participants are effectively receiving information about their progress in relation to a goal and are able to use that information to reflect on whether they also need to recalibrate their goals [20]. However, behavioral interventions do not coach participants on how to articulate their accomplishments per se, so we know little about how accomplishments are articulated and whether how people articulate their accomplishments is associated with better outcomes.

In this study, we examined how participants articulated both their goals and accomplishments in a web-based behavioral weight loss intervention in which all communication occurred in written discussion threads. All participant-reported goals and accomplishments were coded as measurable, repeating, both, or neither. The relationship between the frequency with which participants articulated their goals and accomplishments in these ways and their percent weight loss at 12 weeks was then examined. It was hypothesized that greater sharing goals and accomplishments that were measurable or repeating, particularly those that were *both* measurable and repeating, would be associated with greater percent weight loss than sharing of goals and accomplishments that were neither measurable nor repeating.

## Methods

### Sample

This study is a secondary analysis of data from a previously described randomized feasibility pilot trial of a Facebook-delivered weight loss intervention [21]. Participants were recruited from the Worcester, MA community and were eligible if they used a smartphone, used Facebook, were interested in losing weight, and had a BMI of 25-45 kg/m^2^. Participants were ineligible if they had type 1 or 2 diabetes, were unable to attend assessment visits, had participated in a previous weight loss study with our team, were in a concurrent weight loss program, were taking certain medications, had bariatric surgery, had plans to move during the study period, had a medical condition that would limit their physical activity or diet, did not have clearance from their primary care provider, were pregnant or breastfeeding, did not have a body weight scale at home, or did not speak English.

Eligible participants (N=56) were randomized to 1 of 2 conditions, both of which received a 12-week Facebook-delivered weight loss intervention. However, in 1 group, 3 participants received financial compensation to post in the group daily to be a role model for engagement and social support. The results of the overall trial have been reported previously [16,21]. The 3 participants who received financial incentives were excluded from the present analyses because the content of their posts could have been influenced by the incentives, yielding an analytic sample of 53 participants. As conditions did not differ in weight loss or frequency of participant posts [21], we combined the conditions for the present analyses. The sample size for the parent study was based on the necessities for examining feasibility and acceptability.

### Intervention

All participants received a 12-week Facebook-delivered weight loss intervention based on the DPP Lifestyle Intervention [5]. Two weight loss counselors were responsible for providing counseling to each private, invitation-only Facebook group. Intervention posts were prescheduled to occur twice daily from the counselors’ accounts, and then counselors logged in twice a day to generate discussion, field questions, and provide support and problem-solving to participants [21]. The program was delivered asynchronously, meaning no meeting time was required to participate but rather participants could participate whenever convenient, any time of the day or night. Participants were given a calorie goal to help them lose 1-2 pounds per week and an exercise goal of acquiring at least 175 minutes of moderate-intensity physical activity per week. However, participants were also asked to set small weekly goals for behaviors to work on, report their progress, discuss challenges, and problem solve with the counselors. They were encouraged to set SMART goals and were given instructions on what a SMART goal is. The weight loss counselors were trained to provide corrective feedback, and often participants clarified or specified their goals in a follow-up reply.

### Measures

#### Weight Loss

Baseline and follow-up weights were measured in the lab by research staff using a calibrated balance beam scale. For participants missing weight at follow-up (n=2), we assumed no weight change (baseline value carried forward). The percent weight loss was calculated by dividing the pounds lost between baseline and follow-up by the baseline weight and multiplying by 100.

#### Demographics

At baseline, participants reported demographics on a survey, including age, sex, marital status, and educational attainment.

#### Facebook Posts

Participants’ posts, including original posts and replies to other posts, were extracted from Facebook using Facebook’s application programming interface with a program developed for this purpose.

### Analytic Plan

#### Content Analysis

We previously conducted a directed content analysis of all original posts and replies shared by participants [16]. In the original analysis, all posts were classified as 1 of 10 types (eg, goal and accomplishment) to describe the overall intent of the post. In our original content analysis, posts that included a report of a goal or accomplishment could have been coded as another post type. For example, a post in which a participant shared an accomplishment and a challenge or slip was coded as a challenge or slip post. For this analysis, we re-reviewed participant posts and replies that were originally coded in other categories for the purpose of the previous study. This analysis includes all participant posts with content classified as a goal (ie, reported an intention or plan to make a healthy lifestyle change) or an accomplishment (ie, reported that they completed an action toward a goal). A subset of posts was independently reviewed by a second coder (n=113, 35% of goal posts and n=394, 50% of accomplishment posts). Codes were compared, and discrepancies were resolved through team discussion. Interrater reliability was examined by calculating percent agreement and κ statistics.

##### Goals

Coders classified whether the goals were measurable (ie, specified a particular behavior and how often it will happen; see Table 1). An example of a measurable physical activity goal is “I’m going to walk 3 times this week,” and an example of a specific calorie goal is “I’m going to stay under 1,500 calories each day this week.” Examples of nonmeasurable goals include “I will try harder to eat healthy this week,” “I will increase my willpower,” “I will do my best to stay active,” or any other language that does not specify a specific behavior and how often it will happen. Agreement for measurable goal codes was 81.5% (κ=0.62, 95% CI 0.49-0.78). The 2 coders also determined if a goal was repeating (eg, “I will walk three times a week”) versus a one-time goal (eg, “I will eat a light salad for lunch today”; Table 1). The agreement for repeating codes was 89.7% (κ=0.62, 95% CI 0.49-0.75).

**Table 1:** Participant posts sharing goals and accomplishments in a Facebook-delivered lifestyle intervention by whether posts were phrased in measurable or repeating terms.

Type of post			All posts, n (%)		Participants with any posts, n (%)		Number of posts per participant, median (IQR; range)		Example post
**Goal posts**									
	All goal posts	322 (100.0)		43 (81.1)		4 (1-8; 0-24)		N/A^a^	
	Measurable and repeating	64 (19.9)		28 (52.8)		1 (0-2; 0-8)		“My first exercise goal is to walk for at least fifteen minutes a day this week.”	
	Measurable, not repeating	162 (50.3)		41 (77.4)		2 (1-5; 0-14)		“I’m putting together a shopping list now so that I can get meals prepped this weekend for my lunches and dinners after work.”	
	Not measurable, repeating	16 (5.0)		11 (20.8)		0 (0-0; 0-3)		“...stay away from junk food and hitting the gym more”	
	Not measurable, not repeating	80 (24.8)		34 (64.2)		1 (0-2; 0-9)		“I simply want to feel better mentally and physically. I hope to learn more about nutrition.”	
**Accomplishment posts**									
	All accomplishment posts	789 (100.0)		47 (88.7)		10 (4-24; 0-62)		N/A	
	Measurable and repeating	146 (18.5)		39 (73.6)		2 (0-4; 0-11)		“...I did everything right this week. I walked a lot every day. I [swam] laps one day and took that zumba class. I drank water like it was my job and stayed within my calorie range every day except yesterday.”	
	Measurable, not repeating	287 (36.4)		43 (81.1)		3 (1-6; 0-36)		“I did a mile without stopping. Took about 20 minutes. It’s a start.”	
	Not measurable, repeating	146 (18.5)		36 (67.9)		2 (0-4; 0-11)		“I have been following my plan with healthy eating and walking. Slow and steady wins the race!”	
	Not measurable, not repeating	210 (26.6)		39 (73.6)		3 (0-6; 0-14)		“I’m like a little kid and being told no makes the treat all the more desirable. So that is why I will do a bite or two as a cheat and then walk away!”	

^a^N/A: not applicable.

##### Accomplishments

Coders classified accomplishments as measurable or repeating. Measurable accomplishments specified a particular behavior and how often it occurred. An example of a measurable accomplishment is reporting a daily step total (eg, “Got 10K steps today”), a healthy food selection (eg, “Chose the grilled chicken sandwich for lunch”), or eliminating an unhealthy habit (eg, “Cut out my after dinner snack today”; see Table 1). Agreement for measurable codes was 82.4% (κ=0.65; 95% CI 0.58-0.72). An example of a nonmeasurable accomplishment is “I choose to not get down on myself about bad days” or “I have been going out for walks.” The 2 coders also determined if an accomplishment was repeating (eg, “I was under my calorie goal on five days this week”) or a one-time accomplishment (eg, “I was under my calorie goal today”). Agreement for repeating codes was 86.3% (κ=0.70; 95% CI 0.63-0.76).

#### Analytic Plan

Because the number of each type of goal and accomplishment per participant was not normally distributed, we reported medians, IQRs, and ranges. The relationship between frequency of each post type and percent weight loss with crude and age-adjusted linear regression models was assessed. Because the total number of posts sharing goals or accomplishments of different types was small for some post types (eg, repeating but not measurable goals), the association between posting one or more of each type versus none and percent weight loss was first examined. The association between the number of posts of each type and percent weight loss was then examined. Together, these analyses describe if sharing just 1 goal or accomplishment post of each type (ie, measurable and repeating) is associated with weight loss, as well as specifically how much weight loss is associated with each post of a specific type. Similar to other studies examining the relationship between engagement in a web-based intervention and weight loss [13], we planned to also adjust for gender and race or ethnicity; however, we did not have an adequate distribution in our sample to do so, and thus our adjusted regression model includes only age. All regression models were checked to ensure they met the assumptions of linear modeling. Scatter plots of any posts and number of posts and percent weight loss (ie, a linear relationship) were reviewed. Diagnostic plots were examined to assess the distribution of residuals (ie, normally distributed and homogeneity of variance), and Shapiro-Wilk tests were used to additionally assess whether residuals were normally distributed. We also assessed for the presence of outlying or potentially influential observations. Results from these analyses indicated no clear violations of the model assumptions and adequate model fit. Analyses were conducted using SAS 9.4 (SAS Institute, Inc).

### Ethics Approval

The University of Massachusetts Medical School Institutional Review Board approved this study (H00001484). As this was a pilot trial, with data collection initiated before 2017, it did not meet the Applicable Clinical Trial final rule for ClinicalTrials.gov and was not preregistered.

## Results

### Demographics and General Result

Participants (N=53) were predominantly female (n=48, 91%), non-Hispanic White (n=48, 91%), married (n=35, 66%), and college educated (n=30, 57%). On average, participants were 46.2 (SD 10.5) years old with a baseline BMI of 32.4 (SD 4.8) kg/m^2^. As previously reported, during the 12-week program, participants lost an average of 2.6% ±3.5% (range −12.5% to 5.4%) of their body weight, with 26% (n=14) losing 5% or more of their baseline body weight [19]. Overall, participants made 2918 posts to the Facebook group, with a median of 37 (IQR 16-76; range 0-262) posts per participant. Higher post frequency in general was significantly associated with greater percent weight loss (*r*=−0.38; *P*=.005) [16].

### Goal Posts

#### Overview

During the 12-week intervention, participants posted 322 posts that included a goal, of which 19.9% (n=64) were measurable and repeating, 50.3% (n=162) were measurable but not repeating, 5% (n=16) were not measurable but were repeating, and 24.8% (n=80) were not measurable or repeating. The majority (n=43, 81%) of participants posted at least 1 goal, and participants shared a median of 4 (IQR 1-8; range 0-24) posts that included goals (Table 1). In adjusted models, the frequency of sharing goal posts was associated with weight loss, and each goal post shared was associated with an average of 0.2% greater weight loss (95% CI −0.3% to 0.0%; Table 2).

**Table 2:** Distribution of participant posts sharing measurable or repeating goals and percent weight loss in a Facebook-delivered lifestyle intervention.

Type of goal post	All goal posts, n (%)	Shared 1+ goal posts^a^				Number of goal posts^b^		
		Participants with any goal posts, n (%)	Crude β (95% CI)	Adjusted^c^ β (95% CI)	Number of goal posts per participant, median (IQR; range)		Crude β (95% CI)	Adjusted^c^ β (95% CI)
All goal posts	322 (100.0)	43 (81.1)	−1.5 (−4.0 to 0.9)	−0.7 (−3.0 to 1.7)	4 (1-8; 0-24)		−*0.2* *(−0.4 to −0.1)*^d^	−*0.2* *(−0.3 to 0.0)*
Measurable	226 (70.2)	42 (79.3)	−2.1 (−4.4 to 0.2)	−1.3 (−3.5 to 0.9)	3 (1-7; 0-17)		−*0.3* *(−0.5 to −0.1)*	−0.2 (−0.4 to 0.0)
Not measurable	96 (29.8)	34 (64.2)	−1.6 (−3.6 to 0.3)	−1.1 (−2.9 to 0.7)	1 (0-2; 0-10)		−*0.5* *(−0.9 to −0.1)*	−0.4 (−0.7 to 0.0)
Repeating	80 (24.8)	30 (56.6)	−*2.8* *(−4.5 to −1.0)*	−*2.2* *(−3.9 to −0.4)*	1 (0-2; 0-8)		−*0.7* *(−1.2 to −0.3)*	−*0.5* *(−1.0 to 0.0)*
Not repeating	242 (75.2)	43 (81.1)	−1.5 (−4.0 to 0.9)	−0.7 (−3.0 to 1.7)	3 (1-7; 0-20)		−*0.2* *(−0.4 to −0.1)*	−0.2 (−0.4 to 0.0)
Measurable and repeating	64 (19.9)	28 (52.8)	−*2.6* *(−4.4 to −0.8)*	−*1.9* *(−3.7 to −0.2)*	1 (0-2; 0-8)		−*0.7* *(−1.2 to −0.1)*	−0.4 (−1.0 to 0.2)
Measurable, not repeating	162 (50.3)	41 (77.4)	−1.7 (−4.0 to 0.5)	−0.8 (−3.0 to 1.4)	2 (1-5; 0-14)		−*0.4* *(−0.7 to −0.1)*	−0.2 (−0.5 to 0.0)
Not measurable, repeating	16 (5.0)	11 (20.8)	−*3.1* *(−5.3 to −0.9)*	−*2.5* *(−4.6 to −0.5)*	0 (0-0; 0-3)		—^e^	—
Not measurable, not repeating	80 (24.8)	34 (64.2)	−1.6 (−3.6 to 0.3)	−1.1 (−2.9 to 0.7)	1 (0-2; 0-9)		−*0.5* *(−1.0 to 0.0)*	−0.3 (−0.7 to 0.2)

^a^β represents the difference in mean percent weight loss for participants who shared 1+ goal post versus those who did not share any goal posts.

^b^β represents the difference in mean percent weight loss for each goal post shared.

^c^Adjusted for age.

^d^Italicized entries indicate statistical significance (*P*<.05).

^e^Due to the small sample size, this type of post did not meet the assumptions for the regression model.

#### Measurable Goals

Most participants (n=42, 79%) shared at least 1 post that included a measurable goal. In adjusted models, sharing posts that included measurable goals was not associated with greater weight loss (Table 2).

#### Repeating Goals

More than half of the participants (n=30, 57%) shared at least 1 post that included a repeating goal. In adjusted models, sharing at least 1 post that included a repeating goal was associated with an average of 2.2% greater weight loss (95% CI −3.9% to −0.4%), and each post that mentioned a repeating goal was associated with an average of 0.5% greater weight loss (95% CI −1.0% to 0.0%).

#### Measurable and Repeating Goals

More than half of the participants (n=28, 53%) shared at least 1 post that included a goal that was both measurable and repeating (Table 1). In adjusted models, sharing at least 1 post that included a measurable and repeating goal was associated with an average of 1.9% greater weight loss (95% CI −3.7% to −0.2%; Table 2); sharing each post that mentioned a goal that was measurable and repeating was not associated with greater weight loss.

### Accomplishment Posts

#### Overview

During the 12-week intervention, participants posted 789 posts that included an accomplishment, of which 18.5% (n=146) were measurable and repeating, 36.4% (n=287) were measurable but not repeating, 18.5% (n=146) were not measurable but were repeating, and 26.6% (n=210) were neither measurable nor repeating (Table 1). The majority of participants (n=47, 88%) posted at least 1 accomplishment, and participants shared a median of 10 (IQR 4-24; range 0-62) posts that included accomplishments (Table 1). In adjusted models, the frequency of sharing accomplishment posts was associated with weight loss, where each accomplishment post was associated with an average of 0.1% greater weight loss (95% CI −0.1% to 0.0%; Table 3).

**Table 3:** Distribution of participant posts sharing measurable or repeating accomplishments and percent weight loss in a Facebook-delivered lifestyle intervention.

Type of accomplishment post	All accomplishment posts, n (%)	Shared 1+ accomplishment posts^a^				Number of accomplishment posts^b^		
		Participants with any accomplishment posts, n (%)	Crude β (95% CI)	Adjusted^c^ β (95% CI)	Number of accomplishment posts per participant, median (IQR; range)		Crude β (95% CI)	Adjusted^c^ β (95% CI)
All accomplishment posts	789 (100.0)	47 (88.7)	0.0 (−3.0 to 3.0)	0.2 (−2.6 to 2.9)	10 (4-24; 0-62)		−*0.1* *(−0.2 to 0.0)*^d^	−*0.1 (−0.1 to 0.0)*
Measurable	433 (54.9)	46 (86.8)	0.1 (−2.7 to 2.9)	0.1 (−2.5 to 2.7)	6 (2-9; 0-49)		−*0.1* *(−0.2 to 0.0)*	−0.1 (−0.2 to 0.0)
Not measurable	356 (45.1)	42 (79.3)	−1.7 (−4.1 to 0.6)	−1.2 (−3.3 to 1.0)	4 (2-9; 0-25)		−*0.2* * (−0.4 to −0.1)*	−*0.2 (−0.3 to 0.0)*
Repeating	292 (37.0)	41 (77.4)	−1.8 (−4.1 to 0.4)	−1.9 (−3.9 to 0.2)	4 (1-9; 0-23)		−*0.3* *(−0.4 to −0.1)*	−*0.2 (−0.3 to 0.0)*
Not repeating	497 (63.0)	46 (86.8)	−1.3 (−4.1 to 1.6)	−0.6 (−3.2 to 2.0)	6 (3-13; 0-42)		−*0.1 * *(−0.2 to 0.0)*	−0.1 (−0.2 to 0.0)
Measurable and repeating	146 (18.5)	39 (73.6)	−1.4 (−3.5 to 0.8)	−1.7 (−3.6 to 0.3)	2 (0-4; 0-11)		−*0.3 * *(−0.6 to 0.0)*	−0.2 (−0.5 to 0.1)
Measurable, not repeating	287 (36.4)	43 (81.1)	−1.7 (−4.1 to 0.7)	−0.9 (−3.2 to 1.4)	3 (1-6; 0-36)		−0.1 (−0.3 to 0.0)	−0.1 (−0.2 to 0.0)
Not measurable, repeating	146 (18.5)	36 (67.9)	−*2.0 * * (−4.0 to 0.0)*	−1.7 (−3.6 to 0.1)	2 (0-4; 0-11)		−*0.5 * *(−0.8 to −0.3)*	−*0.4 (−0.7 to −0.2)*
Not measurable, not repeating	210 (26.6)	39 (73.6)	−1.3 (−3.5 to 0.8)	−0.9 (−2.9 to 1.1)	3 (0-6; 0-14)		−*0.3 * *(−0.5 to 0.0)*	−0.2 (−0.4 to 0.1)

^a^β represents the difference in mean percent weight loss for participants who shared 1+ goal post versus those who did not share any goal posts.

^b^β represents the difference in mean percent weight loss for each goal post shared.

^c^Adjusted for age.

^d^Italicized entries indicate statistical significance (*P*<.05).

#### Measurable Accomplishments

Most participants (n=46, 87%) shared at least 1 post that included a measurable accomplishment. In adjusted models, sharing posts that included measurable goals was not associated with greater weight loss (Table 3).

#### Repeating Accomplishments

Most participants (n=41, 77%) shared at least 1 post that included a repeating accomplishment. While sharing at least 1 post that included a repeating accomplishment was not associated with greater weight loss overall, sharing each post that included an accomplishment that was repeating was associated with an average of 0.2% greater weight loss (95% CI −0.3% to 0.0%; Table 3).

#### Measurable and Repeating Accomplishments

Most participants (n=39, 74%) shared at least 1 post that mentioned an accomplishment that was both measurable and repeating (Table 1). In adjusted models, sharing such posts was not associated with greater weight loss (Table 3).

## Discussion

### Principal Findings

Engagement data from web-based weight loss programs that rely on text-based interactions allows us to study how participants discuss their goals and accomplishments. This study provides insights into the specific ways people articulate their goals and accomplishments that may signal the development of habits that promote weight loss. A greater frequency of sharing goals and accomplishments were both independently related to weight loss. In particular, goals that were articulated in either repeating or both measurable and repeating terms were associated with greater weight loss, but for accomplishments, only those articulated in repeating terms were associated with greater weight loss.

In the DPP Lifestyle Intervention, participants are taught to set SMART goals each week [5,18], but variability exists in the extent to which they actually do so. In this study, we found that setting SMART goals and doing so frequently were associated with greater weight loss. Participants who specified parameters about their goals may have been more likely to have had a specific plan in place, which is an important element of goal setting. Goal setting, and specifically setting the context, frequency, duration, or intensity of a goal, has been defined within the Behavior Change Taxonomy as encompassing action planning as well [22]. Indeed, setting specific parameters around a goal is associated with improved outcomes in behavioral interventions. Our findings regarding goals are in line with past research that showed that participants who set physical activity goals that specified the time a goal would be enacted were more likely to follow through with the goal [23]. This may be particularly important for participants with ambitious weight-loss goals. One study found that among people who set large weight loss goals, those with more specific diet and exercise goals lost more weight [24].

These findings suggest several areas for additional research. First, research is needed about how best to support participants in a web-based setting to set SMART goals and how to provide corrective feedback when goals are stated in a way that is neither measurable nor repeating. The weight loss counselors provided corrective feedback; however, participants did not always modify their goal in a reply, though it is possible they may have changed their plan without sharing this in the group. Further, little is known about why participants do not set SMART goals when they are coached to do so. Research is needed to understand reluctance to articulate specific targets in a goal (eg, frequency or duration), as it may be related to low self-efficacy, poor planning skills (eg, executive function deficits), or barriers to accomplishing the goal. As an example of the latter, a participant who feels little control over their schedule may be reluctant to publicly share a measurable and repeating exercise goal because they are not confident that they will be able to accomplish the goal. It may also be that participants set SMART goals but do not share them in the group in a way that articulates the features of SMART. Web-based behavioral interventions (where participants engage in conversation threads) provide an opportunity to flag and intervene upon goals that are not expressed in measurable or repeating terms. Digital tools that assist participants in setting SMART goals also may be useful in web-based behavioral weight-loss programs. Additional content on the merits of SMART goals and research supporting their efficacy may be useful.

In regards to accomplishments, findings revealed that more frequent sharing of accomplishments in general was predictive of weight loss in general, as was sharing of accomplishments expressed in repeating terms (eg, went for a walk every day at lunch last week). This suggests that the expression of repeating accomplishments may signal habit formation [25], an important behavioral milestone associated with long-term weight loss maintenance [26]. Indeed, a growing number of recent studies find that habit, as opposed to intentional choices, contributes more to behaviors affecting energy balance, such as physical activity, diet, and sedentary time [25,27,28]. Further, sharing repeating accomplishments may also be associated with participants more effectively applying feedback when reflecting on their goal progress [20]. This reflection could then lead to a recalibration of goals when necessary [20].

Contrary to our hypothesis, participant posts sharing accomplishments in measurable terms were not predictive of weight loss. In fact, posts sharing accomplishments in *nonmeasurable* terms were predictive of weight loss. We did not coach participants on how to apply the SMART framework to accomplishments in the same way that we did goals. For example, a participant might have accomplished their goal to stay under their daily calorie goal but reported successful execution of this goal more succinctly as “I did well with my eating this week!” Further research should explore how people prefer to articulate their accomplishments in a web-based group setting and the barriers to doing so. Perhaps people who accomplish ambitious goals avoid being overly descriptive of their accomplishments so as not to come off as braggadocios in the group, or it may simply feel like providing all the details of their accomplishment in their post is too time-consuming to type out. People are likely to post their accomplishments to solicit positive reinforcement from the counselor and the group. Thus, great detail about the accomplishment may be felt unnecessary to produce such a response.

In this study, goals and accomplishments were solicited through a variety of posts at different times, so they were not necessarily linked to each other in a way that would show reliably whether a goal was reported later as an accomplishment. On Sunday mornings, participants were asked to report on how they did on their goals each week, and some responded, but others shared at different times or not at all, and this varied from week to week. Future studies could more directly link specific goals to associated accomplishments to examine the extent to which setting a goal and reporting on the outcome are more predictive of weight loss than how goals and accomplishments are articulated.

### Limitations and Strengths

This study has several limitations. First, it is possible that how participants articulated their goals and accomplishments was not exactly how they thought of their goals and accomplishments. For example, a participant could have set SMART goals for themselves but articulated them in the group in a way that was neither measurable nor repeating. However, we did find a relationship between how goals and accomplishments were articulated in the group and weight loss that suggests that what is shared in the group may be a proxy for how they were thinking about their goals and accomplishments. Second, our sample was predominantly female and non-Hispanic White, similar to many behavioral weight loss intervention trials [29,30], and the analyses conducted here were planned post hoc and therefore not taken into account in the original sample size estimation. Combined with our modest sample size, this homogeneous sample limited our ability to control for additional variables in our analyses, such as gender and race or ethnicity. Research is needed with larger and more diverse (eg, racially or ethnically, educationally, and by gender) samples to explore if similar traits of goals and accomplishments are associated with weight loss. Finally, the parent study excluded participants who did not have a body weight scale at home or did not speak English, which reduced the generalizability of the results.

This study also has several strengths. A major strength of this study is the use of objective engagement data to define goals and accomplishments. Content analysis of micro-level engagement data from web-based weight loss programs provides the opportunity to dive deeply into participation in ways that have not previously been available to researchers [31]. While participation and engagement have traditionally been conceptualized as session attendance [32-34], web-based programs that allow people to engage via written exchanges offer a transcript of every conversation that occurred during the intervention. These data can be used to study the nuances of how specific types of engagement impact weight loss. While we focus here on using these data to the specifics of setting goals and reporting accomplishments that are related to weight loss, this method has the potential to be adapted to better understand a range of discussions and post types and their relationships with behavioral and clinical outcomes.

### Conclusions

Much remains to be explored to fully understand not just how much engagement but what types of engagement are associated with better outcomes in web-based behavioral weight loss programs [31]. A deeper understanding of what types of engagement are associated with greater weight loss can help us refine intervention content in ways that solicit these types of engagement from participants. This type of data may also offer guidance to participants on how to engage in ways that may increase their success in web-based lifestyle interventions.

## Abbreviations

DPP: Diabetes Prevention Program
SMART: Specific, Measurable, Action-oriented, Relevant, and Time-bound
